# Retinal Pigment Epithelial Abnormality and Choroidal Large Vascular Flow Imbalance Are Associated with Choriocapillaris Flow Deficits in Age-Related Macular Degeneration in Fellow Eyes

**DOI:** 10.3390/jcm12041360

**Published:** 2023-02-08

**Authors:** Norihiro Nagai, Yasuaki Mushiga, Yoko Ozawa

**Affiliations:** 1 Department of Ophthalmology, St. Luke’s International Hospital, 9-1 Akashi-cho, Chuo-ku, Tokyo 104-8560, Japan; 2 Laboratory of Retinal Cell Biology, St. Luke’s International University, 9-1 Akashi-cho, Chuo-ku, Tokyo 104-8560, Japan; 3 Department of Ophthalmology, Keio University School of Medicine, 35 Shinanomachi, Shinjukuku, Tokyo 160-8582, Japan

**Keywords:** choriocapillaris, OCT angiography, retinal pigment epithelium, asymmetric choroidal vasculature, age-related macular degeneration

## Abstract

Choriocapillaris flow deficits detected on optical coherence tomography angiographs were retrospectively analyzed. In 38 age-related macular degeneration (AMD) fellow eyes, without fundus findings (26 men, 71.7 ± 1.9 years old), and 22 control eyes (11 men, 69.4 ± 1.8), the choriocapillaris flow area (CCFA) ratio and coefficient of variation (CV) of the CCFA ratio (which represented the heterogeneity of the ratio), negatively and positively correlated with age (all *p* < 0.01), respectively. Moreover, the respective mean values were lower (*p* = 0.0031) and greater (*p* = 0.002) in AMD fellow eyes than in the control eyes. The high-risk condition of AMD fellow eyes was defined by a CCFA ratio <58.5%, and the CV of the CCFA ratio ≥0.165 (odds ratio (OR), 5.408; 95% confidence interval (CI): 1.117–21.118, *p* = 0.035, after adjusting for age and sex) was related to the presence of fundus autofluorescence abnormality (OR, 16.440; 95% CI, 1.262–214.240; *p* = 0.033) and asymmetrically dilated choroidal large vasculature (OR, 4.176; 95% CI, 1.057–16.503; *p* = 0.042), after adjusting for age and sex. The presence of fundus autofluorescence abnormality indicated a retinal pigment epithelium (RPE) abnormality. The RPE volume was reduced in the latter eye group, particularly in the thinner choroidal vasculature. In addition to aging, RPE abnormality and choroidal large vascular flow imbalances were associated with exacerbated heterogeneous choriocapillaris flow deficits in AMD fellow eyes without macular neovascularization.

## 1. Introduction

Advanced multimodal imaging techniques allow for the location of subtle changes in the retina and choroid. Using such imaging techniques, the pathogenesis of age-related macular degeneration (AMD), a leading cause of blindness [1,2], has been thoroughly investigated [3,4]. Visual function is irreversibly impaired in both late-stage AMD (characterized by macular neovascularization (MNV)) and/or chorioretinal atrophy. Early AMD is characterized by drusen and/or pigmentary abnormalities [5,6]; hence, investigating possible changes before early AMD is detected would be of value so that early biomarkers for AMD-risk prediction could be established, and so that the fundamental pathogenesis of this condition could be elucidated. This would contribute to the development of new therapeutic approaches.

Choriocapillaris dropout and/or dysregulation have been reported both in the surrounding and outside areas of the MNV lesion in the eyes, in early-, intermediate-, and late-stage AMD. [7,8,9] Consistently, visual function was also reportedly impaired in the surrounding areas [10,11]. Moreover, we previously reported heterogenous choriocapillaris flow deficits in AMD fellow eyes and high-risk eyes [12], in the absence of early- or late-stage AMD findings [13]. As choriocapillaris flow deficits most likely cause relative hypoxia, the deficits would be associated with elevated levels of the vascular endothelial growth factor (VEGF), which is responsible for MNV development [14], and/or with tissue atrophy; therefore, heterogenous choriocapillaris flow deficits found in AMD fellow eyes may represent precocious AMD changes [13] that likely persist and progress after AMD onset and which possibly affect AMD progression and/or recurrence.

Choriocapillaris maintenance is regulated by physiological levels of VEGF that are derived from retinal pigment epithelium (RPE), wherein early AMD changes, such as local lipid accumulation and inflammation, are observed [15]. Conversely, the RPE and photoreceptors are nourished by the choroidal flow, which comes from the posterior ciliary arteries, through the choriocapillaris [16], and which drains via the vortex veins [17]. Asymmetric vortex-vein dilatation is observed in central serous chorioretinopathy (CSC) [18,19] and AMD [20,21], including pachychoroid-related AMD, polypoidal choroidal vasculopathy, and conventional neovascular AMD [21]; however, the relationships between choriocapillaris flow deficits, RPE, and/or choroidal large vascular flow changes remain elusive.

In this study, we analyzed AMD fellow eyes which were at high risk for AMD [12], but which did not indicate that they were in the early or late stages of AMD. This was achieved using multimodal imaging to examine which pathological conditions in the surrounding tissues would be related to heterogenous choriocapillaris flow deficits. With this study, we aimed to deepen the understanding of the fundamentals of AMD pathogenesis and determine very early biomarkers to predict the risk of AMD in the future.

## 2. Materials and Methods

This retrospective study adhered to the tenets of the Declaration of Helsinki and was approved by the St. Luke’s International University Ethics Committee (approval number: 20-R048). All patients provided informed consent for the research use of their data.

### 2.1. Patients

We retrospectively reviewed the medical charts of patients without ocular diseases, such as retinopathy and glaucoma (excluding mild cataracts), and those with unilateral neovascular AMD. AMD was diagnosed using fluorescein angiography (FA) and indocyanine green angiography (IA) at the Vitreo-Retina Division Clinic of the Department of Ophthalmology at St. Luke’s International Hospital in Tokyo, Japan, between April 2020 and January 2022. AMD fellow eyes showing drusen, pigmentary abnormalities confirmed via slit-lamp fundus examinations (performed by a retina specialist (YO)), RPE detachment, choroidal neovascularization, and chorioretinal atrophy (such symptoms were confirmed via FA, IA, optical coherence tomography (OCT), and OCT angiography (OCTA)) were excluded.

### 2.2. Eye Examinations

All patients underwent best-corrected visual acuity (BCVA) measurements based on refraction tests, slit-lamp examinations, and binocular indirect ophthalmoscopy after pupil dilation with 0.5% tropicamide. The axial length was measured using IOLMaster 500 Zeiss (Carl Zeiss Meditec, AG. Jena, Germany).

### 2.3. OCT and OCTA

OCT sectional images and three-dimensional data were obtained with Heidelberg Spectralis OCT (Heidelberg Engineering, Heidelberg, Germany) to evaluate central retinal thickness, central choroidal thickness, the vertical choroidal vascular diameter of the choroidal large vessels at the foveal region (within 500 μm distance from the fovea), and macular volumes. These evaluations were conducted in accordance with the Early Treatment Diabetic Retinopathy Study (ETDRS) grid of retinal layers using built-in software. The RPE thickness was defined as the number of layers from the RPE to the Bruch’s membrane.

OCTA images were recorded, taking pupil dilation into consideration, using spectral-domain OCT (CIRRUS 5000, Carl Zeiss Meditec, AG). Images of a 3 × 3 mm pattern were assessed after excluding projection artifacts; this was achieved using built-in software that applied a validated semiautomated segmentation algorithm to identify the relevant retinal layers, and if necessary, it applied manual corrections to ensure accurate segmentation. The quality index values that were evaluated based on the signal strength of the OCTA images were all >7. The magnification effect of the OCTA images was corrected using Littmann’s method and the modified Bennett formula [22]. After correction, 2.7 × 2.7 mm images of the choriocapillaris slab were analyzed.

OCTA image signals were evaluated by modifying previously reported methods [13]. Briefly, choriocapillaris slab images were binarized using the Phansalkar local binarization threshold; this can account for the variable illumination or contrast between each image [23], and it enables the measurement of the flow signal area with the original ImageJ (National Institutes of Health, Bethesda, MD, USA; available at http://rsb.info.nih.gov/ij/index.html, accessed on 1 February 2022) [24]. The choriocapillaris flow area (CCFA) ratio was defined as the size of the flow signal area to the size of the analyzed area [13]. To evaluate the coefficient of variation (CV) for the CCFA ratio, which represents the homogeneity or heterogeneity of choriocapillaris flow, the binarized images were split into 18 × 18 small images, and the CCFA ratio of each small image was divided by the standard deviation, then averaged [13].

### 2.4. Fundus Autofluorescence (FAF)

Short-wavelength fundus autofluorescence images were acquired with confocal scanning laser ophthalmoscopy (Spectralis HRA + OCT; Heidelberg Engineering). The magnification effect of the images was corrected using Littmann’s method and the modified Bennett formula [22]. After correction, 2.7 × 2.7 mm images were split into smaller 18 × 18 images. Two retina specialists (NN and YO) defined FAF areas as abnormal if FAF intensity was increased or decreased.

### 2.5. FA, IA, and Red Laser Scanning Using an Ultra-Widefield Retinal Imaging System

FA and IA were performed in AMD patients using Spectralis HRA (Heidelberg Engineering), and retinal images were obtained using red laser scanning. The asymmetry or symmetry of choroidal vasculature in the macular region was evaluated through IA images for patients with AMD, and through red-laser images for all patients. If the choroidal watershed zone was deviated by more than one disc diameter, the choroidal vasculature was defined as asymmetrical.

### 2.6. Statistical Analyses

Data are expressed as mean ± standard error. The Mann–Whitney U test, Fisher exact test, multiple logistic regression analysis, and Wilcoxon signed-rank test were performed using SPSS version 27.0 (SPSS Japan, Tokyo, Japan). *p* values < 0.05 were considered statistically significant.

## 3. Results

### 3.1. Participants’ Characteristics

The thirty-eight AMD fellow eyes of thirty-eight patients with unilateral late-stage AMD (26 men; mean age, 71.7 ± 1.9 years; range 50–91 years), and 22 eyes of 22 control individuals with no ocular diseases (excluding mild cataract) (11 men; mean age, 69.4 ± 1.8 years; range, 49–82 years) were included in this study (Table 1). There were no significant differences in terms of age, sex, BCVA, axial length, central retinal thickness, central choroidal thickness, vertical choroidal vascular diameter at the foveal region, presence of asymmetry in the choroidal large vasculature, and the RPE volume at the macular region between the groups; however, the presence of FAF abnormalities was observed in nine (23.7%) of the AMD fellow eyes and none of the control eyes (*p* = 0.011). No patient had high myopia (axial length > 26.5 mm). The quality index values of the OCTA images were comparable between the groups.

### 3.2. Risk of Accelerated Choriocapillaris Flow Deficits in AMD Fellow Eyes and AMD Risk Eyes

**Figure 1:**
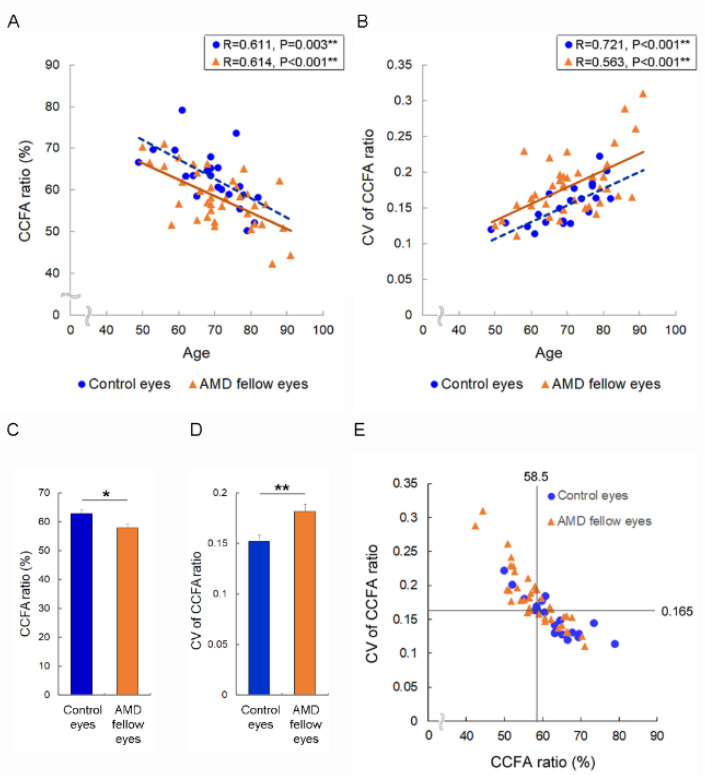
Choriocapillaris flow area (CCFA) ratio and coefficient of variation (CV) of the CCFA ratio of control and age-related macular degeneration (AMD) in fellow eyes. Age was negatively correlated with the CCFA ratio (**A**) and positively correlated with the CV of the CCFA ratio (**B**). The mean CCFA ratio was lower (**C**) and the CV of the CCFA ratio was greater (**D**) in AMD fellow eyes than in control eyes. The cutoff values for the eyes at risk of being AMD fellow eyes are shown in the scatterplot (**E**). * *p* < 0.05, ** *p* < 0.01.

The CCFA ratio and the CV of the CCFA ratio, which represents the heterogeneity of the choriocapillaris deficits [13], were correlated with age in AMD fellow eyes and control eyes (Figure 1A,B); however, the mean CCFA ratio was significantly lower for AMD fellow eyes (58.1 ± 1.0%) than for control eyes (62.9 ± 1.4%, *p* = 0.031) (Figure 1C), and the mean CV of the CCFA ratio was greater in AMD fellow eyes (0.182 ± 0.007) (Figure 1D) than in control eyes (0.152 ± 0.0061, *p* = 0.002). This indicates that choriocapillaris flow deficits progress with age, and the findings were more severe in AMD fellow eyes, even in the absence of MNV or in the early stages of AMD.

To ascertain the factors related to accelerated heterogenous deficits and the imbalance of choriocapillaris flow, independent of aging, we first examined eyes with significant choriocapillaris flow changes. In accordance with the receiver operating characteristic curves (AUC value, 0.697 for the CCFA ratio and 0.723 for the CV of the CCFA ratio), eyes with a CCFA ratio <58.5% and a CV of the CCFA ratio ≥0.165 had a 5.408-fold higher risk of being AMD fellow eyes after adjusting for age and sex (95% confidence interval [CI]: 1.117–21.118, *p* = 0.035) (Figure 1E). We found that the presence of FAF abnormalities, and asymmetry in the choroidal vasculature, conferred a 16.440- and 4.176-fold higher risk (95%CI: 1.262–214.240, *p* = 0.033; 95%CI: 1.057–16.503, *p* = 0.042) of being AMD fellow eyes, respectively, after adjusting for age and sex (Table 2).

### 3.3. Choriocapillaris Flow Deficits in Eyes with FAF Abnormalities

**Figure 2:**
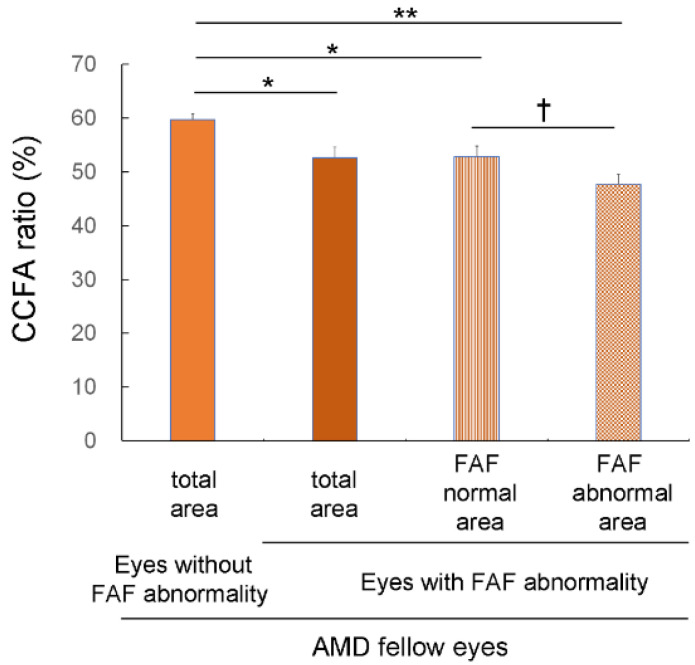
Choriocapillaris flow area (CCFA) ratio of age-related macular degeneration (AMD) in fellow eyes with or without fundus autofluorescence (FAF) abnormalities. In AMD fellow eyes, the CCFA ratio was lower in eyes with FAF abnormalities than in those without, and in AMD fellow eyes with FAF abnormalities, the CCFA ratio was lower in the FAF abnormal area than in the FAF normal area. * *p* < 0.05, ** *p* < 0.01, Mann–Whitney U test. ^†^ *p* < 0.01, Wilcoxon signed-rank test.

In AMD fellow eyes, the CCFA ratio was lower in eyes with FAF abnormalities (52.6 ± 2.0) than in those without (59.7 ± 1.1%, *p* = 0.012, Mann–Whitney U test) (Figure 2). Moreover, in AMD fellow eyes with FAF abnormalities, the CCFA ratio was lower in the abnormal FAF area (47.7 ± 1.9%) than in the normal FAF area (52.8 ± 2.0%, *p* = 0.008, Wilcoxon signed-rank test) (Figure 2 and Figure S1). Notably, in these eyes, the CCFA ratio in the normal FAF area was lower than that of eyes with no FAF abnormalities (*p* = 0.014, Mann–Whitney U test) (Figure 2).

### 3.4. Choriocapillaris Flow Deficits and Their Heterogeneity in Eyes with Asymmetrically Dilated Choroidal Large Vasculature

**Figure 3:**
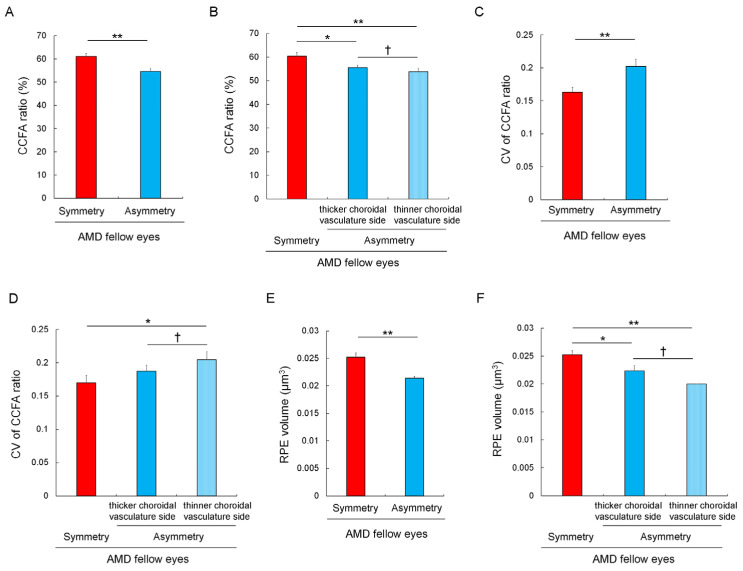
Choriocapillaris flow area (CCFA) ratio, coefficient of variation (CV) of the CCFA ratio, and the retinal pigment epithelium (RPE) volume of AMD fellow eyes with symmetric or asymmetric choroidal vasculature. The total area of the CCFA ratio of AMD fellow eyes with asymmetric choroidal vasculature was smaller than that of those without (**A**), and in AMD fellow eyes with asymmetric choroidal vasculature, the CCFA ratio was lower in the thinner, rather than thicker, choroidal vasculature side (**B**). Both sides exhibited a lower CCFA ratio than the average value of the upper and lower sides of eyes with symmetric choroidal vasculature (**B**). The total area of the CV of the CCFA ratio in AMD fellow eyes with asymmetric choroidal vasculature was greater than that in those without (**C**), and in the AMD fellow eyes with asymmetric choroidal vasculature, the CV of the CCFA ratio was greater in the thinner, rather than, the thicker choroidal vasculature side (**D**). The thinner side exhibited a greater CV of the CCFA ratio than the average value of the upper and lower sides of eyes with symmetric choroidal vasculature (**D**). The average RPE volume of the superior and inferior sides of the inner circle of the ETDRS grid was lower in AMD fellow eyes with asymmetric, rather than symmetric, choroidal vasculature (**E**). In AMD fellow eyes with asymmetric choroidal vasculature, the RPE volume of the area of the thinner choroidal vasculature side was lower than that of the area of the thicker choroidal vasculature side (**F**). Both sides exhibited a lower RPE volume than the average value of the upper and lower sides of eyes with symmetric choroidal vasculature (**F**). * *p* < 0.05, ** *p* < 0.01 with Mann–Whitney U test for comparison between the asymmetry and symmetry groups. ^†^ *p* < 0.05 with Wilcoxon signed-rank test for comparison between the thicker and thinner choroidal vasculature sides in eyes with asymmetric choroidal vasculature.

In AMD fellow eyes, the total area of the CCFA ratio was smaller in eyes with asymmetrically dilated choroidal large vessels (termed asymmetric choroidal vasculature via IA images (Figure S2A)) than in eyes with symmetric choroidal vasculature (54.7 ± 1.3% vs. 61.1 ± 1.4%, *p* = 0.008, Mann–Whitney U test) (Figure 3A). We further analyzed the CCFA ratio of AMD fellow eyes after dividing the OCTA image into upper, middle, and lower areas; one eye with a watershed zone of the choroidal vasculature outside the macular region (i.e., with the thick choroidal vasculature spreading throughout the macula) was excluded because the thicker and thinner choroidal vasculature sides could not be defined. In eyes with asymmetric choroidal vasculature, determining the upper or lower area that comprised the thicker choroidal vasculature side was based on the underlying vascular diameter of the choroidal large vessels (Figure S2A,B). For eyes with symmetric choroidal vasculature, the data concerning the upper and lower areas were averaged.

Consequently, in AMD fellow eyes with asymmetric choroidal vasculature, the mean CCFA ratio was lower in the thinner, rather than the thicker, choroidal vasculature side (53.8 ± 1.4% vs. 55.5 ± 1.2%, *p* = 0.022, Wilcoxon signed-rank test) (Figure 3B). In eyes with asymmetric choroidal vasculature, the mean CCFA ratios of the thicker and thinner choroidal vasculature sides were both lower than the averaged CCFA ratio of the upper and lower areas in eyes with symmetric choroidal vasculature (60.4 ± 1.6%, *p* = 0.031 and 0.008, respectively; Mann–Whitney U test) (Figure 3B).

The total area of the CV of the CCFA ratio was greater in eyes with asymmetric than symmetric choroidal vasculature (0.203 ± 0.011 vs. 0.163 ± 0.007, *p* = 0.005) (Figure 3C). Moreover, in AMD fellow eyes with asymmetric choroidal vasculature, the mean CV of the CCFA ratio was greater in the thinner, rather than the thicker, choroidal vasculature side (0.204 ± 0.012 vs. 0.187 ± 0.009, *p* = 0.027, Wilcoxon signed-rank test) (Figure 3D). The mean CV of the CCFA ratio in the thinner choroidal vasculature side in AMD fellow eyes with asymmetric choroidal vasculature was greater than the average value of the upper and lower areas of eyes with symmetric choroidal vasculature (0.170 ± 0.011, *p* = 0.019; Mann–Whitney U test); the mean CV of the CCFA ratio in the thicker choroidal vasculature side showed a similar trend (*p* = 0.078; Mann–Whitney U test) (Figure 3D).

Taken together, choriocapillaris flow deficits and imbalances were more severe in eyes with asymmetric choroidal vasculature, and the severity was clearer in the thinner choroidal vasculature side.

In AMD fellow eyes, excluding one eye that had a choroidal–vasculature watershed zone outside the macula, we analyzed the RPE volume of the inner macular ring area in accordance with the ETDRS grid (1–3 mm from the fovea) [25,26]; this corresponds with the area that was subject to OCTA analyses (Figure S2C). We found that the average RPE volume of the superior and inferior areas was lower in eyes with asymmetric, rather than symmetric, choroidal vasculature (0.021 ± 0.001 μm^3^ vs. 0.025 ± 0.001 μm^3^, *p* = 0.00065; Mann–Whitney U test) (Figure 3E). Then, in the AMD fellow eyes with asymmetric choroidal vasculature, the superior and inferior inner macular ring areas were defined as either being thinner or thicker choroidal vascular sides, depending on the underlying vascular diameters. We found that the RPE volume was lower in the thinner, rather than thicker, choroidal vasculature side (0.020 ± 0.000 μm^3^ vs. 0.022 ± 0.001 μm^3^, *p* = 0.045; Wilcoxon signed-rank test) (Figure 3F). Moreover, the RPE volumes of the thinner and thicker choroidal vasculature sides were significantly lower than the average RPE volume of the superior and inferior inner ETDRS grids of eyes with symmetric choroidal vasculature (0.025 ± 0.001 μm^3^) (*p* = 0.015 and *p* < 0.001, respectively; Mann–Whitney U test) (Figure 3F). These results indicated that asymmetric choroidal vasculature is related to a lower RPE volume, and this was particularly clear from the thinner choroidal vasculature sides.

## 4. Discussion

We demonstrated that the CCFA ratio decreased and the CV of the CCFA ratio increased with age, both in the control and AMD fellow eyes; however, the mean CCFA ratio was lower, and the mean CV of the CCFA ratio was greater, in AMD fellow eyes than in age-matched control eyes. The condition of the eyes with a CCFA ratio <58.5% and a CV of the CCFA ratio ≥0.165 (which carried a 5.408-fold higher risk of eyes being AMD fellow eyes after adjusting for age and sex) was related to the presence of FAF abnormalities and of asymmetry in the choroidal large vasculature, which caused choroidal watershed deviation. The presence of asymmetry in the choroidal vasculature was related to a reduction in RPE volume, and this was particularly clear in the thinner choroidal vasculature side.

The CCFA ratio and CV of the CCFA ratio were also affected by age in the control eyes, thus suggesting that the choriocapillaris may exhibit age-related frailty and vulnerability, even in the absence of ocular disease. Age-related frailty is identified by reductions in the number of rod [27,28,29] and cone [30,31] photoreceptor cells in the macula, which causes impaired visual function. Visual function is ascertained using a visual electrophysiology system [31], a dark adaptation test [29], and by assessing the macular pigment density [32,33]. Among these parameters, an increased risk for AMD was reported for eyes with low levels of macular pigment density [33,34] and for eyes which exhibited thinning of the photoreceptor segment; this was measured using OCT [35]. For eyes with choriocapillaris flow deficits, further details can be found in our previous study [13]. Choriocapillaris flow deficits could induce relative hypoxia, and thus increase the risk of neovascularization and/or RPE, as well as photoreceptor degeneration, which could induce AMD phenotypes. Given that age is the major AMD risk factor [36], age-related retinal and choriocapillaris frailty may increase susceptibility to developing AMD.

In addition, a combined lower CCFA ratio and greater CV of the CCFA ratio indicates higher levels of heterogenous deficits and an imbalance in terms of choriocapillaris flow; this increases the risk of an eye being AMD, and it increases the risk of AMD fivefold, thus suggesting that choriocapillaris flow changes are further promoted by factors other than age. These factors could be termed second-hit stimuli, which gradually drive the condition closer to disease onset.

For CSC, a “two-hit theory” was proposed; this involves second-hit stimuli, in addition to a susceptible basal condition” [37,38,39]. The basal condition of choroidal venous congestion was stimulated by hyperperfusion that was triggered by other factors, such as sympathetic hyperactivation, thus inducing choroidal overflow; this finally overwhelmed the RPE barrier capacity and led to the accumulation of subretinal fluid. Based on the current findings, heterogenous deficits and an imbalance in terms of choriocapillaris flow may appear with age, and be promoted by factors other than aging, such as smoking, metabolic syndrome, and light exposure, all of which increase the risk of AMD from an epidemiological standpoint [36,40]; the influence of these factors on choriocapillaris flow deficits may involve multiple processes.

To explore the underlying pathological conditions that are affected by second-hit stimuli, eyes with a CCFA ratio <58.5% and a CV of the CCFA ratio ≥0.165 were assessed after adjusting for age and sex. Interestingly, two independent factors arose—the presence of FAF abnormalities and asymmetry in the choroidal large vasculature.

FAF was recorded using blue light (480 nm wavelength), and the dominant source of the signal involved the accumulation of fluorophores as lipofuscin in lysosomal storage bodies in RPE cells; this most likely caused RPE disorders, and a local reduction in terms of signal may have resulted from the absence of local RPE [41,42]. Therefore, alterations in FAF may indicate RPE disorders [41].

FAF abnormalities comprised a risk factor for more severe changes in the CCFA ratio and CV of the CCFA ratio, thus suggesting that RPE abnormalities may be related to choriocapillaris flow deficits. Indeed, FAF abnormal areas showed a more severe reduction in terms of the CCFA ratio, which is consistent with a previous report concerning postmortem eyes with AMD; more than half of the choriocapillaris vascular area that showed a reduction in CCFA ratio was found to have RPE degeneration [43]. Among AMD fellow eyes, an impairment of the CCFA ratio in the total area was more severe in eyes with FAF abnormalities than in those without; although, the FAF abnormal area was relatively small, and it may not explain the reduction in the CCFA ratio of the total area of eyes with FAF abnormalities, thus suggesting that the manner in which FAF changes are detected may not be sufficiently sensitive to show subtle changes in the RPE outside the abnormal FAF area. Moreover, subtle but clear RPE changes may have already existed outside this area in eyes with FAF abnormalities.

**Figure 4:**
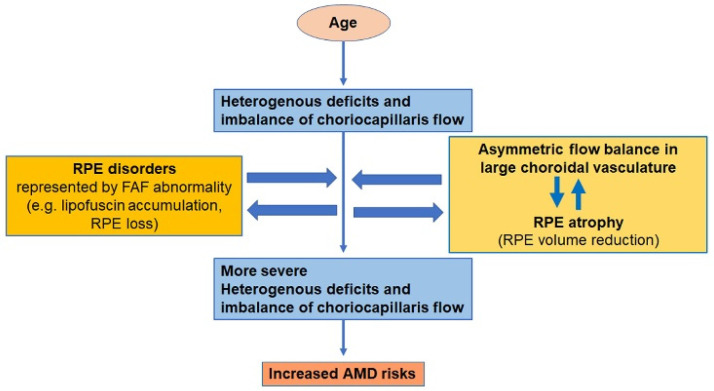
Hypothesis of the progression of heterogenous deficits and imbalanced choriocapillaris flow which increases the risk of age-related macular degeneration (AMD). Choriocapillaris flow deficits progress in a heterogenous manner with age. In addition, retinal pigment epithelium (RPE) changes, as detected by fundus autofluorescence, and in the presence of an asymmetric flow balance in the large choroidal vasculature, reduce the RPE volume measured with optical coherence tomography promote the deficits. Choriocapillaris flow and the RPE condition may interact to advance the heterogenous choriocapillaris flow deficits, which may increase the risk of AMD.

The RPE changes may have caused choriocapillaris flow deficits because RPE-derived VEGF is required for choroidal vasculature and flow [44]. Moreover, the RPE may have also been affected by choriocapillaris flow deficits because the RPE is nourished by the choriocapillaris [16,45]. Both mechanisms may have acted to form a positive feedback loop that exacerbates the heterogenous choriocapillaris flow deficits (Figure 4).

The presence of asymmetry in the choroidal vasculature, first reported in the CSC as dilated choroidal veins extending from the distal end to the ampulla [46,47], was also a risk factor for severe choriocapillaris flow changes. The CCFA ratio and CV of the CCFA ratio were more significantly impaired in AMD fellow eyes with asymmetric choroidal vasculature than in those without. Asymmetry in the choroidal vasculature is reportedly caused by congestion in the choroidal vortex veins [18,37], followed by an increased outflow resistance in the sclera [48,49,50,51]. An imbalance in the large choroidal vascular flow may have affected the balance of the overall choroidal flow, which could cause a reduction in choriocapillaris flow, in a heterogenous manner, as has been shown in the current study (Figure 4). The area of the thinner choroidal vascular side, not the abnormally dilated side, showed more severe deficits and imbalances in choriocapillaris flow. This was in contrast to previous CSC findings, wherein choriocapillaris filling delay was observed in dilated veins [52]; however, these findings were observed in eyes with active CSC, with subretinal fluid, and they might have been subject to the effects of CSC-related second-hit stimuli [37,38,39]. Investigating whether the thinner choroidal vasculature side exhibits the flow steal phenomenon, or whether the thicker side exhibits a choriocapillaris dilatation due to congestion after the remodeling of the choroidal vessels [53,54], could be a future topic of research.

Moreover, the asymmetricity was also related to the RPE volume of the corresponding side (Figure 4). Although we did not include eyes with visible drusen and/or visible RPE atrophy, theoretically, the RPE volume can increase with an increased number of deposits, and it can decrease via atrophic changes. As the RPE volume was lower in eyes with asymmetric choroidal vasculature, particularly in the thinner vasculature side where more severe choriocapillaris flow deficits were observed, a reduction in RPE volume, and RPE atrophy in the presence of asymmetry in the choroidal large vasculature, may be associated with imbalanced choriocapillaris flow deficits; in this case, both directional interactions between the RPE and choriocapillaris would be involved.

Considering all the results, given that the progression of heterogenous deficits and imbalance of choriocapillaris flow, in addition to age-related changes, increased AMD risk. Given that the progression was related to RPE changes with or without asymmetricity in choroidal large vasculature, RPE and choroidal large vascular flow imbalances are involved in processes influenced by second-hit stimuli during AMD development. Epidemiologically reported AMD risk factors other than aging, such as smoking, metabolic syndromes [55] such as hypertension and obesity, and light exposure [56,57,58] can induce inflammation, which affect RPE and choroidal vascular remodeling; although, further studies of the underlying mechanisms are required.

Limitations of this study include the relatively small sample size and retrospective design. The reduction in the CCFA ratio may have derived from decreased signal reflections in the choriocapillaris; this may have been due to deposits found in and around the RPE, in particular, the area containing FAF abnormalities, although there were no visible changes according to the fundus examinations or photographs of the studied eyes.

## 5. Conclusions

In conclusion, choriocapillaris flow was impaired by age, but the change was more obvious in eyes at risk of AMD. The heterogenous choriocapillaris flow deficits, and imbalanced choriocapillaris flow, were related to RPE changes that were represented by FAF changes and asymmetric flow balance in the large choroidal vasculature; this was associated with RPE volume reduction, particularly with regard to the thinner vasculature side. These changes may have interacted in a manner akin to a positive feedback loop, in order to exacerbate the choriocapillaris flow deficits, in addition to the progression of the age-related frailty of the tissue, although further studies are required.

## Figures and Tables

**Table 1:** Characteristics of the control and age-related macular degeneration (AMD) fellow eyes.

	Control Eyes	AMD Fellow Eyes	*p*
*n* (eyes)	22	38	
Age (years)	69.4 ± 1.8 (49–82)	71.7 ± 1.9 (50–91)	0.618
Sex (men; eyes (%))	11 (50.0)	26 (64.8)	0.128
BCVA (logMAR)	−0.05 ± 0.01 (−0.18–0.10)	−0.05 ± 0.02 (−0.18–0.40)	0.202
Axial length (mm)	24.10 ± 0.23 22.46–26.02)	24.10 ± 0.21 (21.80–26.43)	0.908
Central retinal thickness (μm)	223 ± 4 (192−269)	227 ± 4 (177–264)	0.988
Central choroidal thickness (μm)	217 ± 23 (76−535)	224 ± 18 (75–489)	0.914
Choroidal vascular diameter (μm)	172 ± 15 (70−346)	192 ± 13 (55–4381)	0.365
FAF abnormality	0 (0)	9 (23.7)	0.011 *
Asymmetry in choroidal vasculature	7 (31.8)	18 (47.3)	0.183
RPE volume (μm^3^)	0.102 ± 0.002 (0.09–0.14)	0.096 ± 0.003 (0.09–0.13)	0.323

Data are expressed as mean ± standard deviation. Mann–Whitney analyses. BCVA, best-corrected visual acuity; FAF, fundus autofluorescence; RPE, retinal pigment epithelium. The RPE volume was measured in the 3 mm diameter area of the Early Treatment Diabetic Retinopathy Study (ETDRS) grid. * *p* < 0.05.

**Table 2:** Risk factors for eyes with heterogenous deficits and an imbalance of choriocapillaris flow were defined in accordance with the choriocapillaris flow area (CCFA) ratio <58.5% and the coefficient of variation (CV) of the CCFA ratio ≥0.165.

	OR	*p* Value	95% Confidence Interval
FAF Abnormality	16.440	0.033 *	1.262 to 214.240
Asymmetry in choroidal vasculature	4.176	0.042 *	1.057 to 16.503

Multiple logistic regression analysis adjusted for age and sex. CCFA, choriocapillaris flow area; CV, coefficient of variation; FAF, fundus autofluorescence; RPE, retinal pigment epithelium. * *p* < 0.05.

## Data Availability

Data are available upon request of the corresponding author with appropriate reason.

## Supplementary Materials

The following supporting information can be downloaded at: https://www.mdpi.com/article/10.3390/jcm12041360/s1, Figure S1: The choriocapillaris flow area (CCFA) ratio of the abnormal fundus autofluorescence (FAF) area (bordered by blue line) is 47.8% and that of the normal FAF area is 51.5%. Image of an AMD fellow eye of a 68-year-old woman; Figure S2: The watershed zone of the choroidal vasculature is evaluated (yellow dashed line) to determine whether the eye shows watershed deviation related to asymmetricity in choroidal vascular dilatations using indocyanine green angiography (A). Choriocapillaris flow is analyzed using optical coherence tomography angiography (OCTA) images in the upper and lower parts after division into three areas. (B). The retinal pigment epithelium (RPE) volume is analyzed in the upper and lower Early Treatment Diabetic Study (ETDRS) grids at a 1 to 3 mm distance from the fovea (C). Image of an AMD fellow eye of a 68 year old man.
