# Supplementary material for: Retinal Pigment Epithelial Abnormality and Choroidal Large Vascular Flow Imbalance Are Associated with Choriocapillaris Flow Deficits in Age-Related Macular Degeneration in Fellow Eyes
Source: J Clin Med. 2023 Feb 8;12(4):1360. doi: 10.3390/jcm12041360 (PMC9965486; doi:10.3390/jcm12041360)
Supplement: Supplementary file 1 [file jcm-12-01360-s001.zip › jcm-2143476-supplementary.pdf]

Journal of
Clinical Medicine

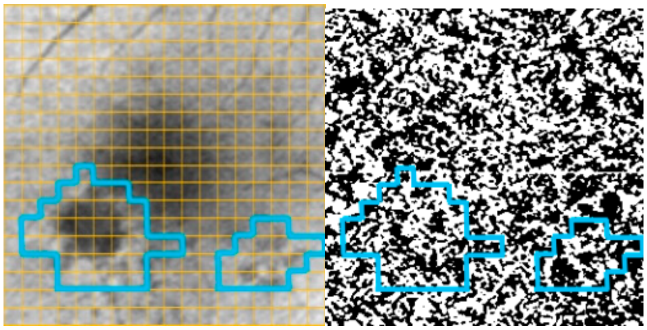

Figure S1. The choriocapillaris flow area (CCFA) ratio of the fundus autofluorescence (FAF) abnormal area (bordered by blue line) is 47.8% and that of the normal FAF area is 51.5%. Image of an AMD fellow eye of a 68 year old woman.

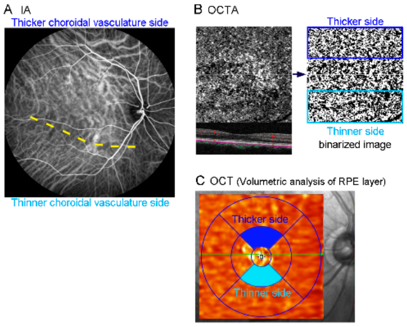

Figure S2. The watershed zone of the choroidal vasculature is evaluated (yellow dashed line) to determine whether the eye shows watershed deviation related to asymmetricity in choroidal vascular dilatation using indocyanine green angiography (A). Choriocapillaris flow is analyzed using optical coherence tomography angiography (OCTA) images in the upper and lower parts after division into three areas. (B). The retinal pigment epithelium (RPE) volume is analyzed in the upper and lower Early Treatment Diabetic Study (ETDRS) grids at a 1 to 3 mm distance from the fovea (C). Image of an AMD fellow eye of a 68 year old man.
